# The role of reactive oxygen species derived from different NADPH oxidase isoforms and mitochondria in oxalate-induced oxidative stress and cell injury

**DOI:** 10.1007/s00240-022-01309-2

**Published:** 2022-02-06

**Authors:** Xiaoyuan Qian, Weisong Wu, Henglong Hu, Xiao Yu, Shaogang Wang, Jianning Zhu, Jiaqiao Zhang

**Affiliations:** 1 Department of Urology, Tongji Hospital, Tongji Medical College, Huazhong University of Science and Technology, 1095 Jiefang Avenue, Wuhan, Hubei China; 2 The Central Hospital of Wuhan, Tongji Medical College, Huazhong University of Science and Technology, Wuhan, Hubei China

**Keywords:** Oxalate, Urolithiasis, Reactive oxygen species, Mitochondria, NADPH oxidase

## Abstract

Hyperoxaluria is a risk factor for urolithiasis and can cause renal epithelial cell injury secondary to oxidative stress. Reactive oxygen species (ROS) produced during cell damage originate from different sources and play different roles. Here, we explored the potential sources of ROS production and investigated the role of ROS from various sources in oxalate-induced oxidative stress and cell injury in normal rat kidney-52 epithelial (NRK-52E) cells. Oxalate-induced injury was assessed by lactate dehydrogenase (LDH) release experiments. 2,7-dichlorodihydrofluorescein diacetate and mitoSOX Red were used to determine the intracellular and mitochondrial ROS (mtROS) production, respectively. The expression level of Nox4, Nox2, and p22 protein was detected by Western blotting to observe the effect of oxalate on nicotinamide adenine dinucleotide phosphate oxidase (NADPH) oxidase (Nox). Furthermore, a specific NADPH oxidase subtype inhibitor and targeted mitochondrial antioxidants were used to preliminarily identify the role of ROS from different sources in renal tubular epithelial cell injury induced by oxalate. We found that oxalate inhibited cell viability, induced LDH release, and prompted intracellular and mitochondrial ROS (mtROS) production. Oxalate also decreased the protein expression level of Nox4 and increased the protein expression level of p22. Mitochondria were also a source of ROS production. In addition, Nox2 inhibitor or mtROS scavenging prevented oxalate-induced cell injury, reversed by an inhibitor of Nox4/1. We concluded that ROS from different sources might play different roles in oxalate-induced renal tubular epithelial cell injury. We also identified new potential targets for preventing or alleviating oxalate-induced renal tubular epithelial cell injury.

**Graphic abstract:**

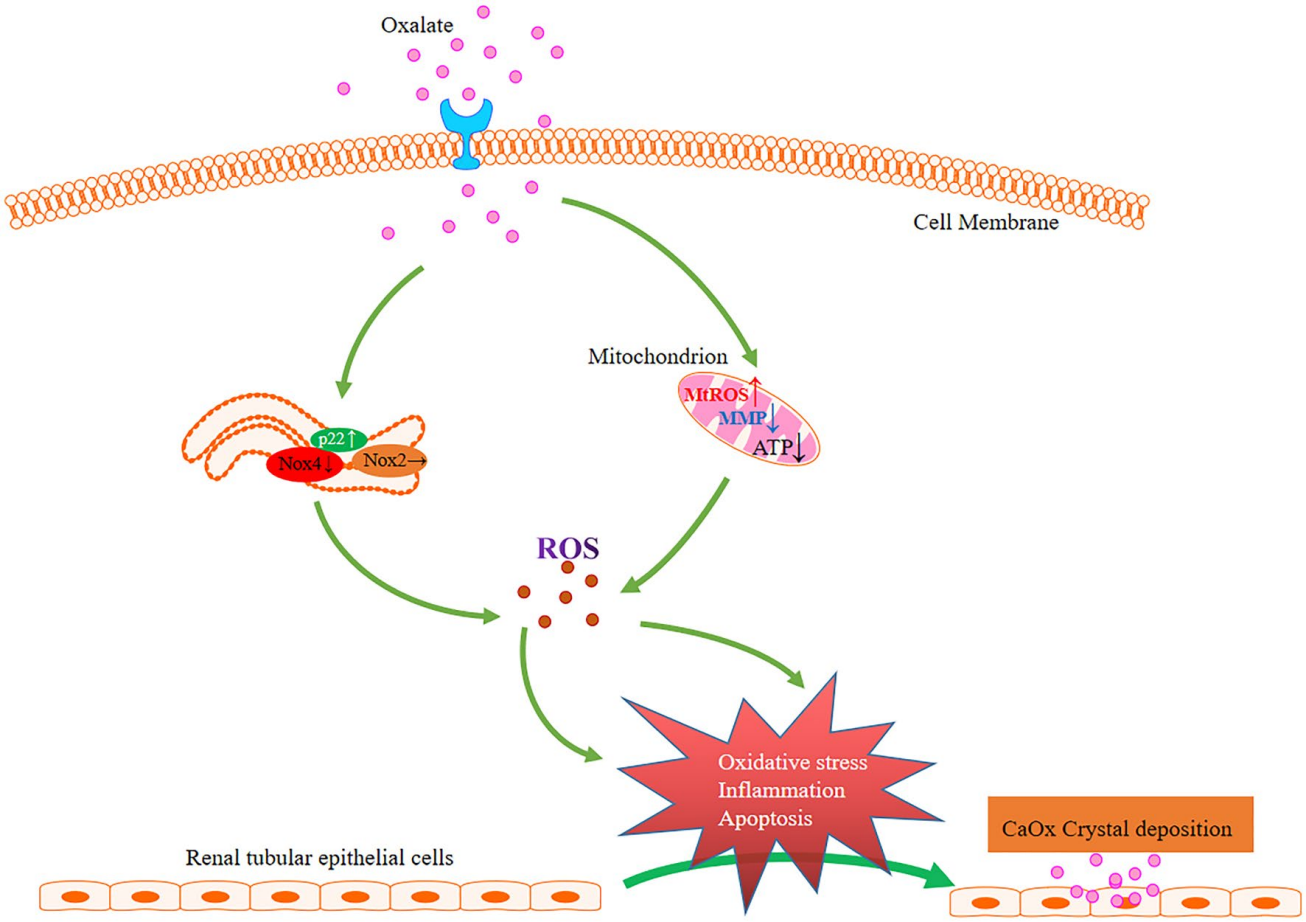

## Introduction

Calcium oxalate calculi are the most common type of urinary calculi, accounting for approximately 70–80% of all cases. Hyperoxaluria is considered one of the cardinal risk factors for urinary stone formation [1, 2]. Exposure to high oxalate (Ox) and/or calcium oxalate (CaOx) crystals can cause injury to renal tubular epithelial cells, and their deleterious effect is closely associated with the generation of reactive oxygen species (ROS) and the activation of nicotinamide adenine dinucleotide phosphate (NADPH) [3, 4]. Cellular responses to the injury induced by Ox or CaOx crystals can be inhibited by antioxidants as well as classical NADPH oxidase (Nox) inhibitors, such as diphenylene iodonium (DPI) [2, 5, 6]. Another classical NADPH oxidase inhibitor, apocynin, has also been verified to protect against renal injury and decrease stone formation in animal models of hyperoxaluria [7–9].

NADPH oxidase is an important source of ROS in the kidney and is involved in several physiological and pathological responses [10, 11]. Thus, it has been considered an important therapeutic target for oxalate-mediated renal tubular epithelial cell injury and stone formation [12, 13]. However, certain aspects related to its role in Ox or CaOx crystal-induced renal tubular epithelial injury remain unresolved. First, DPI and apocynin, the two widely used classical NADPH oxidase inhibitors, have shown a non-specific action towards Nox in previous studies [14, 15]. More importantly, the Nox family comprises seven isoforms, and the expression of Nox4, Nox2, and Nox1 was found to be decreased gradually in the kidney [10, 16, 17].

Since different Nox isoforms have shown different characteristics with respect to their components, expression, subcellular localization, and pattern of production of ROS [10, 18–20], we hypothesized that Nox isoforms may play different roles in renal epithelial cells exposed to oxalate and may be implicated in urinary stone formation. Additionally, mitochondria are also an important site of ROS generation, and oxalate-induced renal tubular epithelial cell injury is related to mitochondrial dysfunction [21, 22]. As with Nox isoforms, the role of mitochondrial ROS (mtROS) in renal tubular cells exposed to oxalate remains unexplored and warrants further research.

In our study, we determined the different sources of ROS in renal proximal tubular epithelial cells and verified their role in oxalate-induced oxidative stress and cell injury. We attempted to locate the sources of ROS (Nox isoforms or mitochondria) that can potentially be targeted to prevent oxalate-induced kidney injury and renal stone formation.

## Materials and methods

### Reagents

Dulbecco’s Modified Eagle’s Medium (DMEM) was purchased from Grand Island Biological Company (Grand Island, NY, USA). Gp91ds-tat (Nox2 inhibitor), GKT37831 (Nox4/1 inhibitor), and MitoTEMPO (mitochondrial antioxidants) were purchased from Sigma-Aldrich (St. Louis, MO, USA). Cell counting kit-8 (CCK-8) was acquired from Signalway Antibody LLC (Maryland, USA). Lactic dehydrogenase (LDH) cytotoxicity assay kit, 2,7-dichlorodihydrofluorescein diacetate (DCFH-DA), and JC-1 dye were obtained from Beyotime Institute of Biotechnology (Jiangsu, China). MitoSOX Red was purchased from Invitrogen Molecular Probes (Carlsbad, CA, USA). Nox4 antibodies were supplied by Abcam (Cambridge, MA, USA). p22 antibodies were obtained from Santa Cruz Biotechnology (Santa Cruz, CA, USA). Finally, Nox2 and β-actin antibodies were purchased from Boster Biological Technology (Wuhan, China).

### Cell culture and treatment

Cell lines of the normal rat proximal tubular epithelium (NRK-52E) were purchased from the National Collection of Authenticated cell Cultures (Shanghai, China). Cells were seeded in DMEM, and 10% fetal bovine serum (Gibco, Grand Island, NY, USA) was added. Next, the cells were cultured in a humidified atmosphere with 5% CO_2_ at 37 °C. The NRK-52E cells were grown in 6-,12- or 96-well plates for 24 h. All cells adherent to the wall were maintained in DMEM, with and without oxalate at different concentrations (0 μmol/L, 100 μmol/L, 300 μmol/L, 500 μmol/L, 700 μmol/L, and 1000 μmol/L) for different durations, and the changes in cell viability and extent of damage were examined to determine the treatment time and concentration of oxalate needed to induce cell damage. Next, cells pretreated with or without gp91ds-tat, GKT37831, and MitoTEMPO for different durations were cultured with DMEM containing 700 μmol/L of oxalate.

### Assessment of cell viability (CCK-8)

The effect of oxalate on cell viability and proliferation was accessed by a CCK-8 assay, according to the manufacturer’s manual. Cells at a concentration of 2 × 10^3^/well were planted in 96-well plates and incubated overnight. Following pretreatment with or without GKT137831(a dual inhibitor of both Nox1 and Nox4) or gp91 ds-tat (an inhibitor of Nox2) for 1 h, the cells were treated with or without oxalate (700 μmol/L) for 24 h. After treatment, the previous media was discarded, and 100 μL of DMEM media containing 10% CCK-8 reagent was replenished in each well. After cells were incubated for 2–3 h, the optical density (OD) at 450 nm was measured. Finally, cell viability was calculated as the ratio of the OD value in the treated group divided by that in untreated controls, expressed as a percentage.

### Lactate dehydrogenase assay

Oxalate-induced injury to cells was detected by measurement of LDH content in media. Cells were planted in 96-well plates at a concentration of 2 × 10^3^/well for 12 h and then inoculated in a serum-free medium and each stimulus was given in groups. Before treatment, they were replaced with serum and sodium pyruvate-free DMEM media. Upon the completion of treatment, the sample was centrifuged, and the supernatant was collected for further testing, according to the manufacturer's instructions. The OD value at 492 nm was obtained.

### Measurement of intracellular ROS production by flow cytometry

DCFH-DA was used to determine intracellular ROS production. Cells were first planted in a 12-well plate and stimulated with oxalate with or without 1 h of pre-incubation with GKT137831 or gp91 ds-tat. After being treated for 3 h, cells were incubated in a serum-free DMEM media containing 10 μM DCFH-DA for 20 min. Then, the cells were collected and washed with 200 μL of serum-free media. Intracellular ROS production was detected by flow cytometry with excitation and emission wavelengths set at 488 nm and 525 nm, respectively.

### Detection of mitochondrial membrane potential (MMP) by fluorescence microscopy

An MMP assay kit (JC-1) was used to detect MMP. Cells were grown in 6-well plates for subsequent detection. Following pretreatment with or without MitoTEMPO for 1 h, the cells were treated with or without oxalate (700 μmol/L) for 3 h. Subsequently, JC-1 working solution was added to cells and maintained for 20 min at 37 ℃ in the dark. After washing with JC-1 staining buffer, cells were observed under a fluorescence microscope (Olympus IX73, Japan). To detect the JC-1 monomer exhibiting green fluorescence, the excitation light was set to 490 nm and the emission light to 530 nm. To detect JC-1 polymer exhibiting red fluorescence, the wavelength of excitation light should be 525 nm and that of the emission light, 590 nm.

### Measurement of mtROS levels by flow cytometry

mtROS production was detected using MitoSOX Red mitochondrial superoxide indicator. After the same steps for detecting intracellular ROS levels were followed, cells were incubated with a 5-μM solution of MitoSOX Red reagent for 10 min at 37 °C in the dark. The cells were then collected, washed, and resuspended in Hanks’ Balanced Salt Solution. The mtROS level was measured by flow cytometry (excitation and emission at 510 and 580 nm, respectively).

### Western blotting analysis

Cells were washed and lysed with radio-immunoprecipitation assay (RIPA) lysis buffer containing a protease inhibitor to extract the total protein. An equal concentration of protein was separated by 8% sodium dodecyl sulfate–polyacrylamide gel electrophoresis and transferred to polyvinylidene fluoride membranes. After being blocked in tris-buffered saline with tween^®^ 20 (TBST) containing 5% non-fat dry milk for at least 1 h at room temperature, the membranes were incubated with specific primary antibodies (1:1000 for rabbit anti-Nox2; 1:1000 for rabbit anti-Nox4; 1:500 for mouse anti-p22; and 1:1000 for mouse β-actin) for at least 16 h at 4 ℃. Next, the membranes were washed three times and incubated with a peroxidase-conjugated secondary antibody (1:10,000 for anti-rabbit or anti-mouse IgG), for 1 h. Finally, the bands were developed using an enhanced chemiluminescence (ECL) western blot detection system (Thermo Fisher Scientific), and the relative protein expression was quantified using Image Lab 6.0 software (Bio-Rad, Hercules, CA, USA) by the gray value.

### Statistical analyses

All continuous data with a normal distribution are shown as mean ± standard deviation (mean ± SD). One-way analysis of variance (ANOVA) followed by Tukey’s post hoc test was used to identify statistically significant associations between variables. A two-sided *P* < 0.05 was considered to be statistically significant. The statistical data were analyzed and graphically represented using GraphPad Prism 7 statistical software (GraphPad Software, Inc., CA, USA).

## Results

### Oxalate promoted intracellular ROS generation

**Fig. 1:**
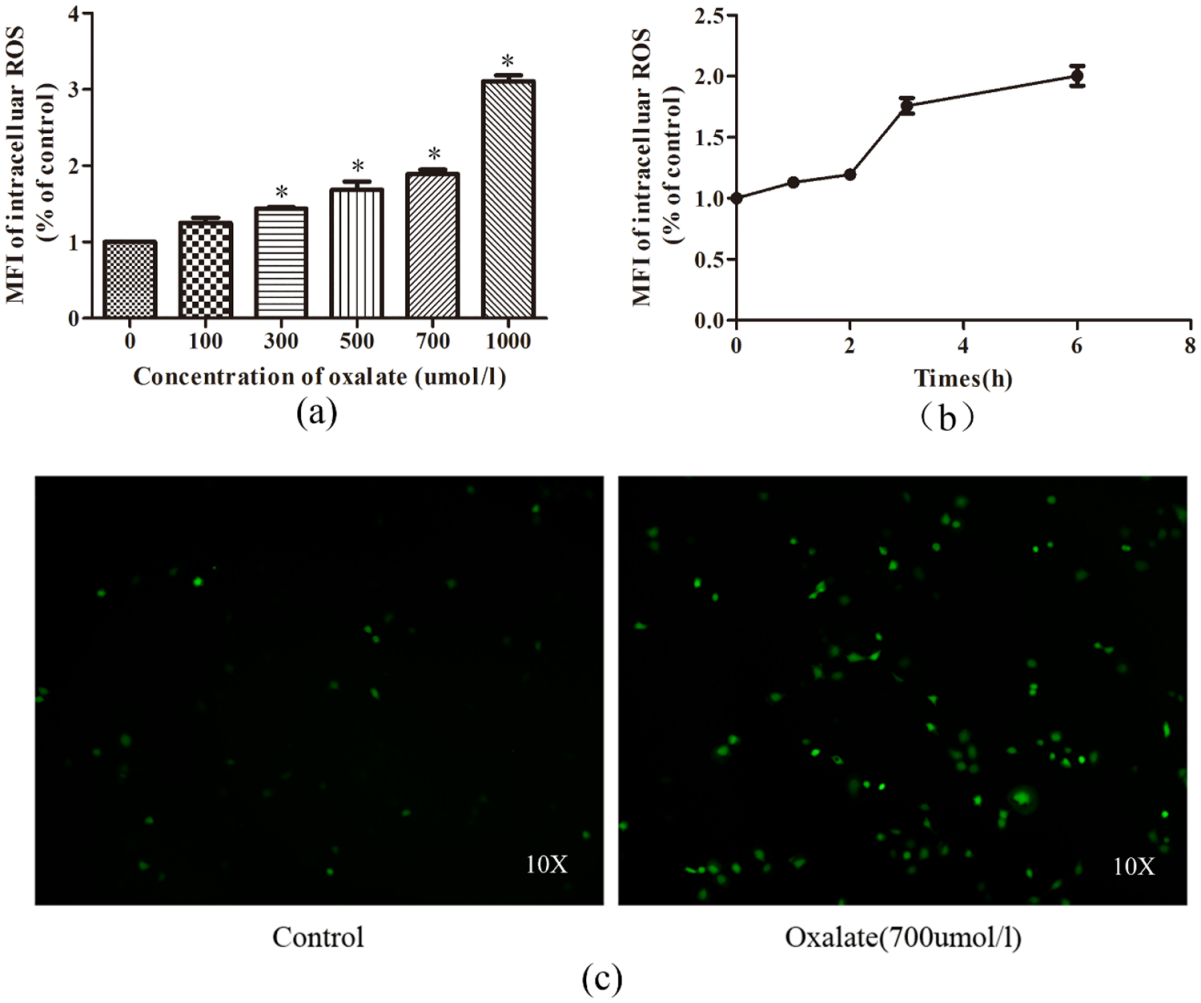
Oxalate-induced intracellular reactive oxygen species (ROS). A 2,7-dichlorodihydrofluorescein diacetate (DCFH-DA) fluorescent probe was applied to detect intracellular ROS. Intracellular ROS levels were estimated by the fluorescence intensity of DCFH, analyzed by flow cytometry (**a**, **b**). **a** Intracellular ROS levels in NRK-52E cells treated with different concentrations (0–1000 μmol/L) of oxalate for 3 h. **b** ROS levels at different time points in NRK-52E cells exposed to oxalate (700 μmol/L). **c** The intracellular ROS levels in the control group and NRK-52E cells treated with oxalate (700 μmol/L) for 3 h were observed under a fluorescence microscope. *P < 0.05 versus the control group

DCFH-DA fluorescence performed to investigate the total intracellular ROS levels in NRK-52E cells showed that relative to the control group, the total ROS production increased in a concentration-dependent pattern in cells with elevated oxalate concentrations (Fig. 1a). Moreover, at an oxalate concentration of 700 μmol/L, the generation of ROS increased gradually in a short time (1–6 h), and ROS production increased substantially at 3 h (Fig. 1b). In addition, under the fluorescence microscope, ROS production was greatly increased in cells treated with oxalate (Fig. 1c).

### Oxalate-induced changes in mitochondrial membrane potential and mtROS generation

**Fig. 2:**
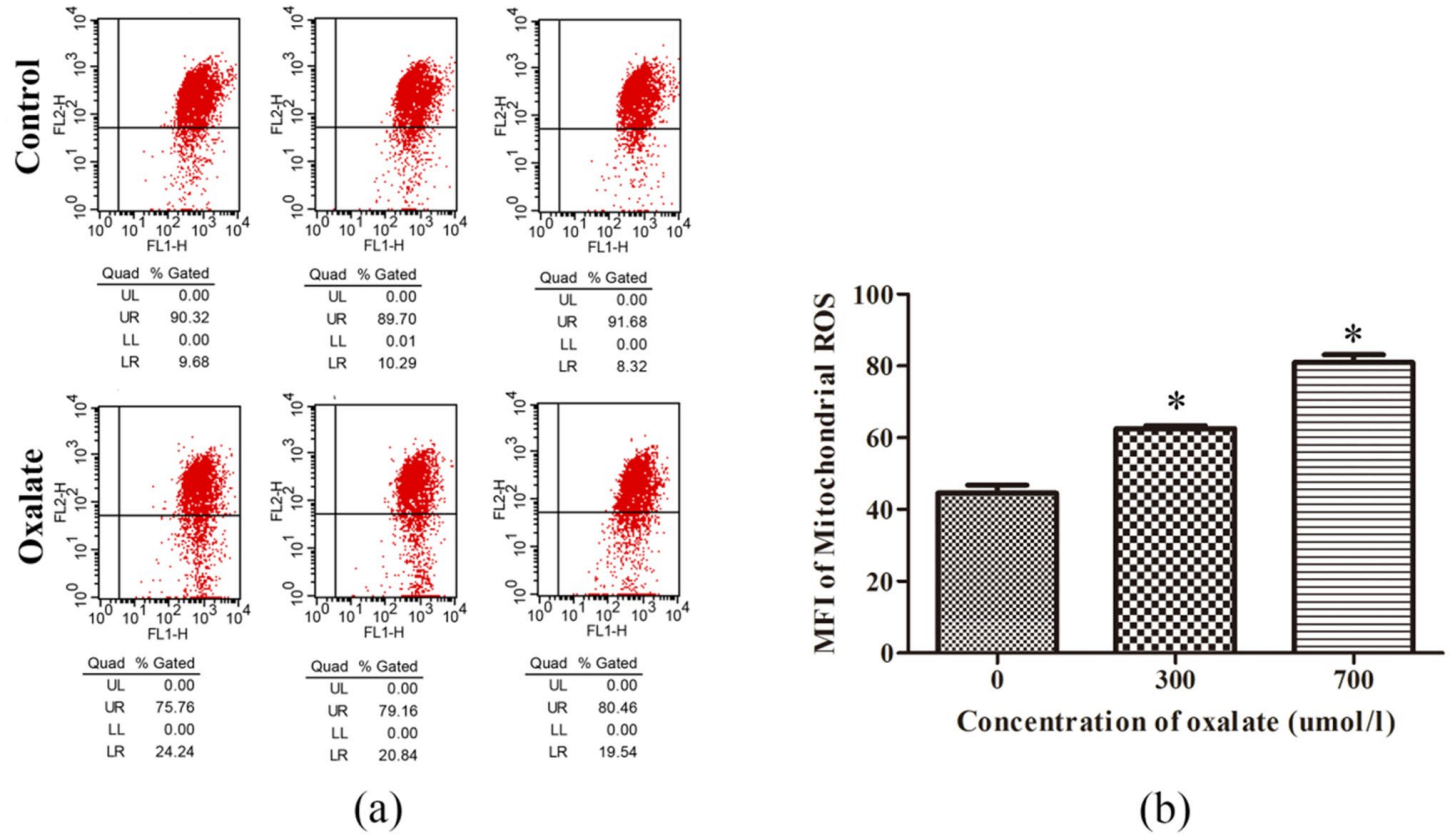
Oxalate-induced changes in mitochondrial membrane potential and mitochondrial ROS production in NRK-52E cells. **a** After NRK-52E cells were treated with oxalic acid (700 μmol/L) for 3 h, the mitochondrial membrane potential was measured by JC-1 using flow cytometry (UR stands for JC-1 polymer; LR stands for JC-1 monomer). **b** The Mito-SOX Red fluorescent probe was used to check for the mtROS production after NRK-52E cells were exposed to oxalate (700 μmol/L) for 3 h and the fluorescence intensity was quantitatively determined by flow cytometry. *P < 0.05 versus the control group

After treatment with oxalate (700 μmol/L) for 3 h, the mitochondrial membrane potential of NRK-52E cells decreased distinctly compared to the control group (Fig. 2a). Further, different oxalate concentrations (300 μmol/L and 700 μmol/L) induced mtROS production in NRK-52E cells. The production of mtROS evidently increased in tandem with the concentration of oxalate (Fig. 2b).

### Effect of oxalate on the expression of Nox subunits

**Fig. 3:**
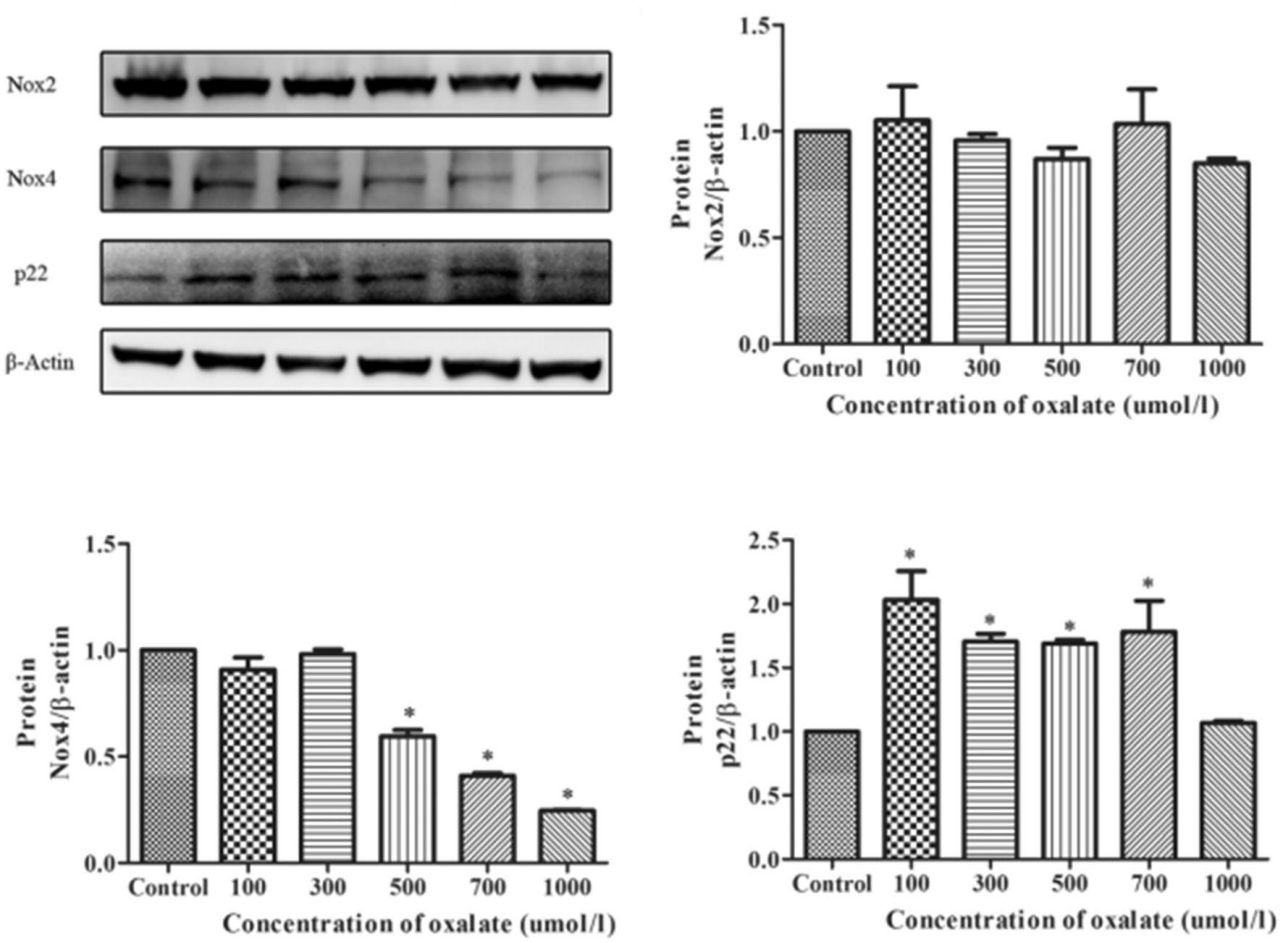
The protein expression of NADPH oxidase subunits in NRK-52E was stimulated by different oxalate concentrations (0–1000 μmol/L) for 24 h. Compared with the control group, oxalate treatment significantly decreased the protein expression of Nox4 and increased that of p22 significantly. However, the protein expression of Nox2 was not particularly affected after oxalate treatment. *P < 0.05 versus the control group

Oxalate can reportedly affect the transcription or protein expression of some subunits of NADPH oxidase [3, 23]. In this study, we examined the expression of the main subunits—p22, Nox2, and Nox4—in NRK-52E cells exposed to oxalate. The expression of Nox4 protein was significantly down-regulated in a concentration-dependent manner, while that of p22 protein was remarkably up-regulated. However, no obvious change was detected in the expression of Nox2 protein (Fig. 3).

### Gp91ds-stat and MitoTEMPO attenuated oxalate-induced oxidative damage

**Fig. 4:**
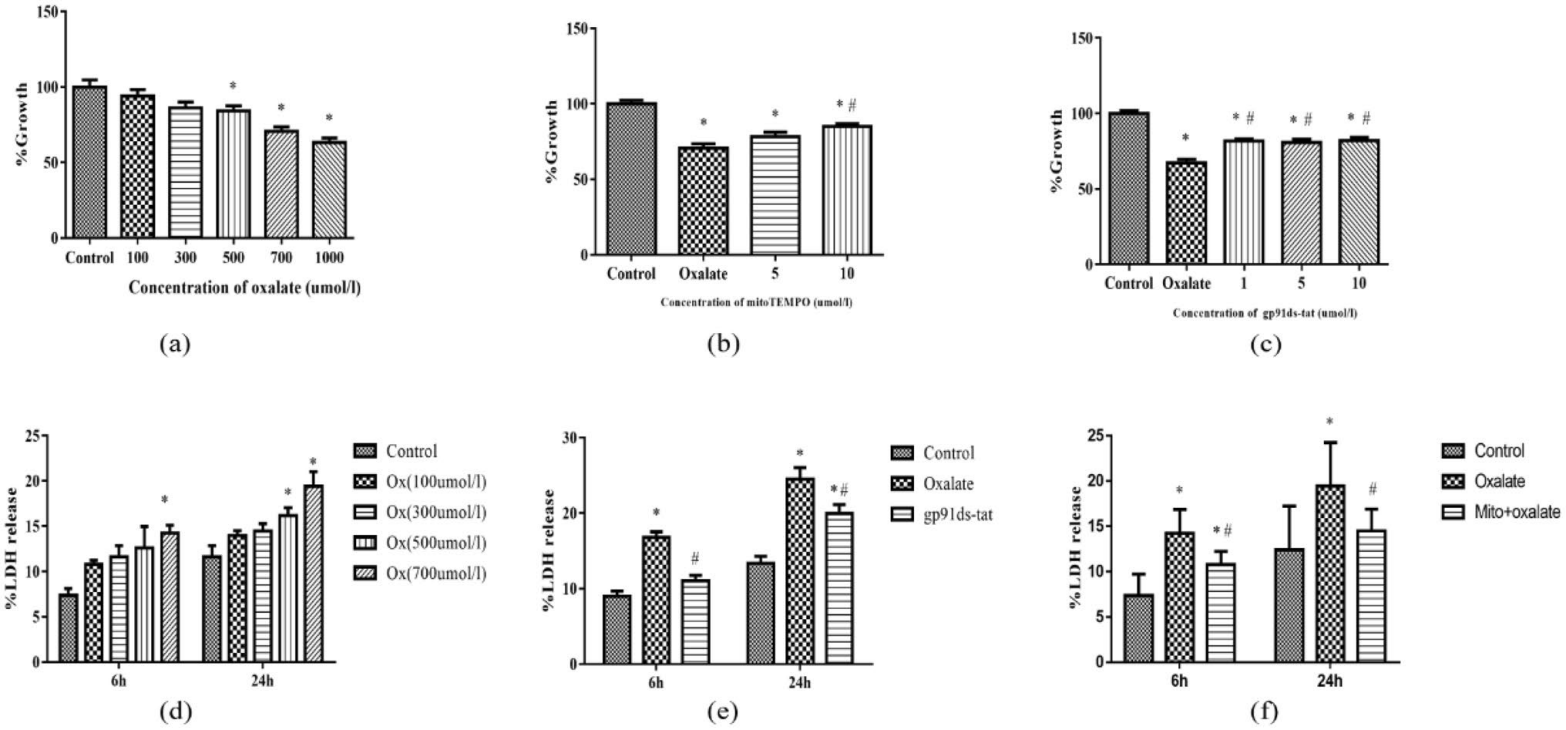
Effects of oxalate, Nox2 inhibitor (gp91ds-stat), and mitochondrial antioxidants (MitoTEMPO) on NRK-52E cell viability and injury. NRK-52E cell viability and injury were evaluated by a CCK-8 assay and LDH assay, respectively. **a** Cell viability was detected post-exposure to in different concentrations of oxalate (0–1000 μmol/L). **b** Compared with that in the oxalate (700 μmol/L, 24 h), mitoTEMPO (10 μmol/L, preincubation for 1 h) significantly increased the viability of NRK-52E cells. **c** On exposure of NRK-52E cells to oxalate (700 μmol/L) for 24 h with or without preincubation with different concentrations of gp91ds-tat (1, 5, and 10 μmol/L) for 1 h, gp91ds-tat (at all concentrations) significantly increased the viability of NRK-52E cells. **d** Intracellular release of LDH was detected when cells were exposed to oxalate at different concentrations (0–1000 μmol/L). **e**, **f** On exposure of NRK-52E cells to oxalate (700 μmol/L) for 24 h with or without preincubation with different concentrations of gp91ds-tat (10 μmol/L) and mitoTEMPO (10 μmol/L) for 1 h, LDH release was measured. *P < 0.05 compared with the control group; ^#^*P* < 0.05 compared with the oxalate group

To explore the effects of oxalate, gp91ds-stat, and Mito TEMPO on the viability of NRK-52E cells, a CCK-8 assay was applied. Oxalate stimulation (0–1000 μmol/L) for 24 h resulted in a decrease in cell proliferation in a dose-dependent manner, and cell viability was significantly inhibited at a high concentration of oxalate (Fig. 4a). Based on the above results, oxalate at a concentration of 700 μmol/L was used in follow-up experiments. After NRK-52E cells were pretreated with a Nox2 inhibitor (gp91ds-stat) and mitochondrial antioxidants (MitoTEMPO) for 1 h, cell viability was significantly reversed (Fig. 4b, c).

To further clarify the damaging effect of oxalate on NRK-52E cells, we tested LDH release activity. Cell injury was significantly increased in a dose-dependent manner after cells were treated with oxalate (100–700 μmol/L), and a similar trend was observed at varying intervals (6 h and 24 h). LDH activity was significantly increased at 24 h after treatment when compared with 6 h (Fig. 4d). On stimulation of NRK-52E cells with oxalate (700 μmol/L) for 24 h with or without preincubation with different concentrations of gp91ds-stat and Mito TEMPO for 1 h, the intracellular release of LDH was significantly decreased in the presence of gp91ds-stat and Mito TEMPO (Fig. 4e, f).

### GKT37831 aggravated oxalate-induced oxidative damage

**Fig. 5:**
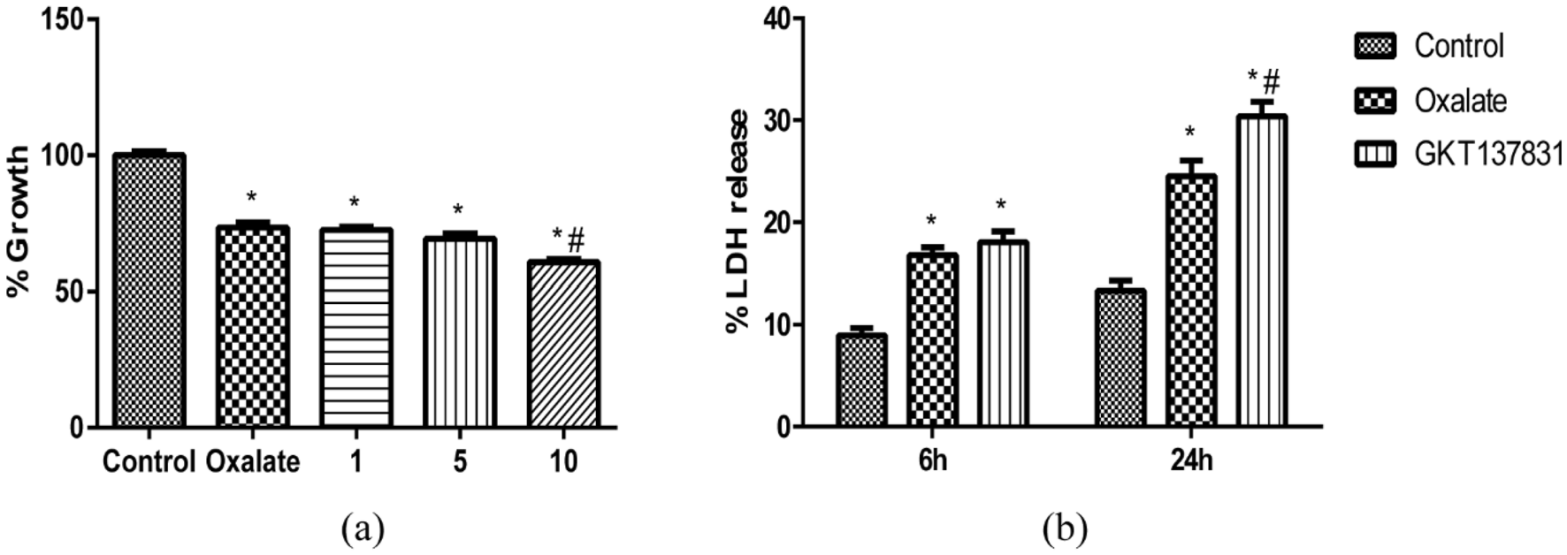
Effect of Nox4/1 or Nox2 inhibitors on oxalate-mediated cell viability and injury. **a** After NRK-52E cells were stimulated with oxalate (700 μmol/L) for 24 h with or without preincubation with different concentrations of GKT137831 (1, 5, and 10 μmol/L) for 1 h, cellular activity was measured. **b** After NRK-52E cells were exposed to oxalate (700 μmol/L) for 24 h with or without preincubation with GKT137831 (10 μmol/L) for 1 h, LDH release was measured. *P < 0.05 compared with the control group; ^#^*P* < 0.05 compared with the oxalate group

Similarly, to verify alterations in NRK-52E cell viability and injury after they were pretreated with Nox4/1 inhibitor (GKT37831), CCK-8 and LDH assays were performed. After preincubation with GKT37831 (10 μmol/L) for 1 h, NRK-52E cell viability was overtly inhibited (Fig. 5a), and the release of LDH became apparent (Fig. 5b).

## Discussion

Hyperoxaluria, defined as an increase in the urinary oxalic acid content, is one of the important risk factors for urinary calculi. Under normal circumstances, the body’s 24-h oxalic acid secretion ranges from 10 to 40 mg, with secretion greater than more than 45 mg being defined as clinical type hyperoxaluria [24]. Depending on the cause, hyperoxaluria is divided into primary, secondary, and idiopathic types [12]. Experimental studies, including cell- and animal-model experiments, have shown that oxalic acid can inflict renal tubular epithelial cell damage, closely related to the activation of NADPH oxidase, ROS generation, and oxidative stress. The injurious effects of oxalic acid have also been effectively reversed by antioxidants and NADPH oxidase [3, 9, 25, 26].

ROS is a general term for a class of reactive molecules and free radicals and mainly includes superoxide anions, nitric oxide radicals, hydroxyl groups, and hydrogen peroxide [13]. The physiological concentration of ROS, especially in specific intracellular organelles is of great significance in maintaining the normal signal and function of cells [15, 27]. However, the generation of excessive ROS can disrupt reduction reactions and result in oxidative stress, thereby leading to pathological effects. Therefore, in terms of their effect on the organism, ROS are divided into two categories—those inducing pathological effects and those mediating physiological effects. However, this classification is inaccurate and constitutes extensive overlap. There are several types and sources of ROS in cells. Furthermore, different types of ROS derived from the same source may play different roles in different biochemical reactions and different subcellular structures [28]. However, it is not clear what role ROS from different sources exert in oxalate-induced renal tubular injury, renal injury, or nephrolithiasis. If one can distinguish between pathological and physiological ROS, selective intervention for pathological ROS would be valuable.

NADPH oxidase is a special enzyme, with its only function being the generation of ROS; other functions have not yet been discovered in recent studies. Depending on the core subunit, it consists of seven subtypes—Nox1–Nox5, Duox1, and Duox2. Meanwhile, p22 is the universal binding subunit of Nox1–Nox4 isoforms [10]. These subtypes further differ in their structural composition, types of reactive oxygen species acted upon, and activation methods. In the kidney, Nox4 and Nox2 are the main subtypes, while Nox1 is also expressed. Therefore, our research mainly focused on Nox4 and Nox2 subtypes. Recent studies on mtROS indicate that it is directly involved in the release of inflammatory factors, which may be a feature of pathological ROS. Our study aimed to preliminarily explore the role of ROS from these sources in oxalate-mediated renal tubular epithelial injury.

Our experiments showed that when NRK-52E cells were exposed to oxalate, the total ROS in the cells and ROS in the mitochondria increased significantly and inhibited cell viability, thereby suggesting their role in the mechanism of cellular injury.

Previous studies have reported that the action of Ox or CaOx crystals on renal tubular epithelial cells is accompanied by the activation of NADPH oxidase, which may be accompanied by changes in the transcription level of relevant subunits [3]. Consistent with this finding, our results demonstrated that the protein expression of each subunit of NADPH oxidase significantly changed after the cells were treated with oxalate. While Nox4 expression was significantly reduced, Nox2 expression was not significantly changed.

The total NADPH oxidase activity in the cell reportedly increases in response to oxalate [3, 23]. p22 binds to its core subunit Nox in Nox2 and Nox4 enzymes. By factoring in the above with our conclusions, we can speculate that the increase in total NADPH oxidase activity is closely related to the significant upregulation of p22 protein expression, and p22 may also be used as a potential therapeutic target.

Further, we used GKT137831 and gp91ds-tat to clarify the role of the Nox subtype in oxalate-mediated renal tubular injury. GKT137831 is a dual inhibitor of Nox1/4, while gp91ds-tat is a Nox2 inhibitor [15]. Our results showed that when Nox1/4 activity was blocked, cell damage was aggravated. This also illustrated that when oxalate acted on NRK-52E cells, the ROS derived from the Nox1/4 enzyme mainly mediated physiological effects and the ROS was blocked to further aggravate pathological damage. Therefore, Nox2 inhibitors play a protective role, and the ROS derived from Nox2 is likely to be a pathological ROS, promoting cell damage.

We also found that oxalate-induced increase of mtROS was accompanied by mitochondrial dysfunction, which may be because mitochondria are not only the source of ROS but also its target [29]. After cells were pretreated with mitoTEMPO, oxalate-mediated cell damage was also reduced. This also verified that mtROS were pathological ROS, and antioxidants targeting mtROS could potentially help reduce cell damage.

While our work offered substantial evidence on the role of ROS derived from Nox isoforms and mitochondria in oxalate-mediated oxidative stress and cell injury, there were a few limitations that should be acknowledged. First, we did not observe the effects of other enzymes, such as xanthine oxidase and decoupled nitric oxide synthase, that can generate ROS [30]. Second, only a few subunits of NADPH oxidase were determined, and the specific species of ROS were not tested. Third, the specific mechanisms by which blockade of ROS derived from different sources affected oxalate-induced cytotoxicity were not elucidated. Furthermore, although the expression of Nox1 in the kidney was relatively low, we did not confirm its expression in cells, which ought not to be ignored in research.

In summary, the present study substantiated that ROS generated from different sources played different roles in renal tubular epithelial cells cultured with oxalate. Interfering with specific sources of ROS may aggravate or reduce cell damage. Most studies on oxalate-induced renal damage have targeted NADPH oxidase. Our study helped identify more specific targets (including Nox2 or Nox4, p22 subunit, and the mitochondria). Based on these targets, new research directions can be followed for the prevention and treatment of oxalate-mediated renal tubular epithelial injury, renal tissue injury, and renal calculi by drug or gene editing.

## Conclusion

We demonstrated that ROS from Nox4 played a protective role in oxalate-induced renal tubular epithelial cell injury, while ROS from Nox2 or mtROS could aggravate cell injury induced by oxalate. These findings may enable the discovery of a new therapeutic target against oxalate-induced renal tubular epithelial cell injury, which can be beneficial for the prevention of renal stones.

## Data Availability

The data supporting the findings of this study are available from the corresponding author upon reasonable request.

## Abbreviations

ROS: Reactive oxygen species
LDH: Lactate dehydrogenase
mtROS: Mitochondrial ROS
NADPH: Nicotinamide adenine dinucleotide phosphate
Nox: NADPH oxidase
Ox: Oxalate
CaOx: Calcium oxalate
DPI: Diphenylene iodonium
CCK-8: Cell counting kit-8
DCFH-DA: 2,7-Dichlorodihydrofluorescein diacetate
DMEM: Dulbecco’s modified Eagle’s medium
OD: Optical density
MMP: Detection of mitochondrial membrane potential
RIPA: Radioimmunoprecipitation assay
TBST: Tris-buffered saline with tween^®^ 20
